# Gender Differences in Body Appreciation and Its Associations With Psychiatric Symptoms Among Chinese College Students: A Nationwide Survey

**DOI:** 10.3389/fpsyt.2022.771398

**Published:** 2022-02-17

**Authors:** Zi-Han Liu, Hong Cai, Wei Bai, Shou Liu, Huanzhong Liu, Xu Chen, Han Qi, Teris Cheung, Todd Jackson, Rui Liu, Yu-Tao Xiang

**Affiliations:** ^1^Unit of Psychiatry, Department of Public Health and Medicinal Administration, Institute of Translational Medicine, Faculty of Health Sciences, University of Macau, Taipa, Macao SAR, China; ^2^Centre for Cognitive and Brain Sciences, University of Macau, Taipa, Macao SAR, China; ^3^Institute of Advanced Studies in Humanities and Social Sciences, University of Macau, Taipa, Macao SAR, China; ^4^Department of Public Health, Medical College, Qinghai University, Xining, China; ^5^Department of Psychiatry, Chaohu Hospital of Anhui Medical University, Hefei, China; ^6^The National Clinical Research Center for Mental Disorders, Beijing Key Laboratory of Mental Disorders Beijing Anding Hospital, The Advanced Innovation Center for Human Brain Protection, School of Mental Health, Capital Medical University, Beijing, China; ^7^School of Nursing, Hong Kong Polytechnic University, Kowloon, Hong Kong SAR, China; ^8^Department of Psychology, University of Macau, Taipa, Macao SAR, China

**Keywords:** body appreciation, gender differences, depressive symptoms, anxiety symptoms, suicidality

## Abstract

**Background:**

Body appreciation (BA hereafter), which reflects approval, acceptance, and respect for one's body while also rejecting media-promoted appearance ideals as the only form of human beauty, is an important aspect of positive body image. Much of the BA literature has been conducted on samples from Western nations but less is known about BA or its correlates in Asian cultural contexts wherein concerns with body image are also common. Toward addressing this gap, we examined gender differences in BA and its associations with common psychiatric symptoms (i.e., depressive symptoms, anxiety symptoms, and suicidality) within a national college student sample from China.

**Method:**

This cross-sectional, nationwide study was conducted between December 27, 2020, and January 18, 2021, based on snowball sampling. Aside from measures of demographics and background factors, Chinese versions of the Body Appreciation Scale-2 (BAS-2), Patient Health Questionnaire (PHQ-9), Generalized Anxiety Disorder Scale-7 (GAD-7), and a standard item on suicidal ideation and planning were administered to assess BA, depressive and anxiety symptoms, and suicidality, respectively.

**Results:**

In total, 2,058 college students (665 men, 1,393 women) in China were assessed. An analysis of covariance revealed that the men had a significantly higher average BA level than did women [*F*_(1,2058)_ = 13.244, *P* < 0.001, Cohen's d = 0.193]. Hierarchical multiple regression analyses revealed BA was negatively associated with symptoms of depression, anxiety, and suicidality within the entire sample (depressive symptoms, β = −0.129, *P* < 0.001; anxiety symptoms, β = −0.101, *P* < 0.001; suicidality, OR = 0.788 *P* = 0.020) and among women (depressive symptoms, β = −0.172, *P* < 0.001; anxiety symptoms, β = −0.131, *P* < 0.001; suicidality, OR = 0.639 *P* = 0.001) but not men.

**Conclusion:**

Chinese college women reported lower BA than their male peers did. Furthermore, among women but not men, elevations in BA corresponded with protective mental health experiences including lower levels of depressive symptoms, anxiety symptoms and suicidality. Findings underscore the potential utility of including BA in mental health assessments of Chinese college students, especially women. Findings also provide foundations for continued research on interventions to increase BA among at-risk young women in China.

## Introduction

Gender differences have been an important ongoing focus of body image research (1) in light of differences in biological, psychological and sociocultural influences that contribute to body image experiences of women vs. men (2, 3). Historically, much of this literature has focused on gender differences in facets of negative body image such as body dissatisfaction, appearance evaluations, appearance investment, discrete body image affect (e.g., anxiety, shame), and clinical eating disorders. In general, girls and women experience more overall body dissatisfaction, investment in and concerns with physical appearance, negative body-related emotions, and clinical eating disorders related to weight/shape than boys or men do (4–8).

More recently, researchers and clinicians have begun to explore gender differences in positive body image. Empirically, measures of negative body image and positive body image typically have significant, inverse correlations (9–11). However, formulations and measures of positive body image are not simply the flipside of negative body image (e.g., lower levels of negative body image). Instead, positive body image is a multidimensional construct that involves appreciation, acceptance and love of one's body, broadly conceptualizing beauty, adaptive appearance investment, emphasis on the body's functional capacities not just its appearance, recognition of inner positivity, and information filtering in a body-protective manner (12). Positive body image indices also explain unique variance in outcomes beyond the impact of negative body image measures. For example, body appreciation (BA), which refers to acceptance, respect, and appreciation of one's body while also rejecting media-promoted appearance ideals as the only form of human beauty (10), has clear incremental validity in scale development research (9). Specifically, studies have found that, even after first controlling for the impact of negative body image measures (i.e., appearance evaluations, body dissatisfaction), BA explained significant, unique, additional variance in criterion measures of intuitive eating, disordered eating, self-esteem, optimism and proactive coping.

Aside from empirical data that illustrate the “added value” of positive body image constructs such as BA to the body image literature, there are important conceptual and practical reasons for their inclusion. Without question, understanding negative body image is critically important for reducing disturbances in eating and body image, accompanying emotional distress, and impairment. However, focusing exclusively on eliminating negative body image symptoms without any consideration of human strengths that protect against body image disturbances and foster flourishing severely curtails efforts to promote health, improve well-being, and prevent or treat body image disturbances. As Tylka reported (13), when deficit-based approaches based on negative body image are the exclusive focus of research and treatment, the best possible outcome is that the patient no longer hates her/his body. Unfortunately, the patient does not love her/his body either and is merely tolerating it. Incorporating positive body image constructs such as BA within the field aids in helping patients to appreciate, respect, love, and honor their bodies in tandem with reducing dysfunction.

During the past two decades, researchers have focused increasingly upon identifying facets of positive body image on which there are gender differences in addition to adaptive and less adaptive experiences that contribute to these gender differences. Ultimately, such data may help to guide the development of tailored interventions to improve positive body image and reduce suffering within and across genders. For example, in relation to BA, a recent meta-analysis based on 40 studies (14) found that males had significantly higher mean BA levels than females did though the effect size for this difference was small (Cohen's d = 0.26). While this review provided preliminary evidence for gender differences in BA, more than 80% of the samples were from Western countries. Hence, findings may not apply to people in non-Western countries where much of the world's population is concentrated. For example, only one included study (15) assessed respondents from mainland China, a nation that comprises over 20% of the global population. Wang et al. (15) found comparatively higher BA scores among boys in their sample of adolescents. However, He et al. (14) found age and sample type moderate gender differences in BA; effect size differences were larger in adolescent samples from middle/high schools than college student or adult community samples. Together, these data highlight the need for studies of college students in non-Western countries such as China. Hence, this study was designed, in part, to assess gender differences in BA in a national sample of Chinese college students.

Although BA is also hypothesized to protect against negative physical and mental health outcomes, comparatively few studies have examined its associations with common psychiatric disturbances including depressive symptoms and anxiety symptoms. Elevations in BA have been linked to significantly lower levels of depressive symptoms (16–18) and anxiety symptoms (18, 19) in samples from Western countries. Nonetheless, the degree to relations between BA and psychiatric symptoms are stronger or weaker *within* samples of women vs. samples of men is unclear because most relevant studies were based solely on women (18–21) or did not assess within gender correlations at all when both women and men were included (16). In the only study to consider associations between BA and depression separately for women vs. men, correlations were significant within each gender (17). Furthermore, in non-Western contexts such as China, links between BA and psychiatric symptoms have been ignored altogether because the preponderance of related research has focused on negative body image (22–26).

Despite significant associations of BA with reduced psychiatric symptoms (depressive, anxiety) that increase risk for suicide (27) researchers have also neglected potential links between BA and suicidality (e.g., suicidal thoughts, plans, attempts). This gap is notable given that suicidality is prevalent within frequently studied populations such as college students (28–31) and has significant associations with measures of negative body image (5, 32–34). As well, because people with higher levels of BA are more likely to pursue health behaviors that protect the physical body (e.g., cancer screening, sun protection, healthy eating, vaccinations) (16, 35–37), it is plausible that BA is also potentially protective against suicidality. Once again, however, it is not clear whether the strength of this relationship is similar for college women vs. college men.

Based on the preceding overview, this study has two main purposes, each of which addresses gaps in the BA literature. First, although studies on gender differences in BA have proliferated at a rapid rate, only one study, to date, was conducted in mainland China (15). Because that study examined gender differences on BA among adolescents, results may not apply to college student cohorts, which tend to have weaker average effect size differences (14). Hence, the initial purpose of this study was to test the hypothesis that Chinese college men would also report significantly more BA, on average, than Chinese college women would, in line with the broader literature.

Second, as documented above, higher levels of BA correlate with lower levels of depressive and anxiety symptoms as well have increased engagement in life-sustaining, health protective behaviors that are discordant with self-harm or suicidality. Once again, however, associated studies have been limited to Western samples and have not typically compared strengths of these relations within samples of women vs. samples of men. In response, we assessed the extent to which BA was associated with symptoms of depression, anxiety and suicidality for Chinese college students in general as well as within each gender. Based on evidence of inverse relations in related work, we hypothesized that elevations in BA would be related to lower levels of reported depressive symptoms, anxiety symptoms and suicidality. However, given the lack of research comparing patterns of within gender relations, no a priori hypotheses were generated regarding strengths of association among college women vs. college men.

## Methods

### Setting and Sample

This was a cross-sectional study conducted among Chinese college students from 25 provinces (in total, China has 34 provinces) between December 27, 2020, and January 18, 2021, using a snowball sampling method. Following previous published studies (38–40), an online questionnaire was designed with the QuestionnaireStar application and distributed through WeChat, a widely used social communication application with more than 1 billion users in China (41). Influential academic staff including University Presidents, Faculty Deans and Department heads forwarded the online questionnaire to their teaching staff and encouraged them to participate in data collection. The following selection criteria were used: (1) undergraduate students; (2) ethnic Chinese; (3) ability to understand the purpose and content of the assessment; (4) no major medical or psychiatric condition. To reduce missing data in the questionnaire, all questions required a response to proceed to subsequent items, though participants could also withdraw from the study at any point if they chose. Teachers who assisted in recruitment permitted eligible participants (18 years and older) to proceed directly by completing an online written informed consent. Conversely, students under 18 years of age were also required to contact and provide an online written informed consent from a legal guardian before proceeding. This study was approved by the Institutional Review Board (IRB) of Beijing Anding Hospital.

### Measures

#### Body Appreciation

BA was assessed with the 10-item Body Appreciation Scale-2 (BAS-2), Chinese version (10, 42). The BAS-2 has 10 items, each of which is rated on a 5-point Likert scale (i.e., 1 = never, 2 = seldom, 3 = sometimes, 4 = often, 5 = always). Item scores are summed and averaged to obtain an overall BA score with higher scores reflecting greater BA. The BAS-2 Chinese version has satisfactory psychometric properties with Cronbach alphas of α = 0.89 and α = 0.86, respectively, among Chine college women and men (42). BAS-2 alpha coefficients were excellent in the current sample (women: α = 0.93; men: α = 0.94).

#### Psychiatric Symptoms

##### Depressive Symptoms

The Patient Health Questionnaire (PHQ-9), Chinese version, assessed severity of depressive symptoms (43–45). Each of its nine items is scored from 0 (not at all) to 3 (nearly every day) with higher scores indicating more severe depressive symptoms. The cut-off value is ≥ 5 for classifying one as “having depression” (43). The Chinese version of PHQ-9 had satisfactory psychometric properties with a Cronbach's α as 0.85 (46). In this sample, PHQ-9 alpha coefficients were satisfactory (women: α = 0.89; men: α = 0.92).

##### Anxiety Symptoms

The Generalized Anxiety Disorder Scale-7 (GAD-7)-Chinese version was used to assess anxiety symptoms; the scale consists of 7 items, each of which is scored from 0 (not at all) to 3 (nearly every day) with higher total scores indicating more severe anxiety symptoms (47, 48). A cut-off value of ≥ 10 is used to classify one as “having anxiety” (47). The Chinese version of GAD-7 had satisfactory psychometric properties with a Cronbach's α as 0.898 (47). GAD-7 alphas in this sample were excellent (women: α = 0.92; men: α = 0.95).

##### Suicidality

Suicidality was assessed with the question, “Have you ever thought about or had a plan to commit suicide?” Respondents were given a dichotomous response (yes/no) option.

#### Demographics and Background Measures

Participants were asked their gender (“female” or “male” as choices), age, academic grade (first to fifth year), and residence (urban vs. rural). Because BA has been linked to having a college major in related work (49), academic major (health-related vs. other) was also assessed. Given significant inverse associations have been found between BA and body mass index (BMI) in both genders (50, 51), height and weight data were assessed to calculate BMI (kg/m^2^).

Previous studies found that both body image and psychiatric symptoms have had significant associations with perceived health status, fatigue and pain (52–58). Therefore, a single item measure was used to assess perceived health status (poor/fair vs. good). Following a recent published study (58), severity of fatigue was assessed with the well-validated single-item Numeric Fatigue Rating Scale (FNRS), Chinese version (59). FNRS scores ranged from “0” (no suffering from fatigue) to “10” (unbearable suffering from fatigue). Current pain severity was examined with a one-item numeric Pain Rating Scale (NPRS) that included “0” (no pain) and “10” (worst pain imaginable) as anchors (60). The NPRS—Chinese version has been widely used in different populations (61–63).

### Design and Data Analysis

Data were analyzed using the IBM Statistical Package for Social Sciences (SPSS) program, version 23.0. A P-P Plot was used to test the normality of continuous variables. To compare gender differences on demographic/background measures, chi-square tests, independent two-sample t tests and Mann-Whitney U Tests were used, as appropriate. An analysis of covariance (ANCOVA) was conducted to compare BAS-2 score between women and men after controlling for the potential differences on other demographic/background measures in this sample. Spearman correlation analyses were performed among all continuous variables (i.e., BAS-2, PHQ-9, GAD-7, age, grade, pain, fatigue, BMI) within each gender to test for collinearity.

For the entire sample and within each gender, hierarchical multiple linear regression analyses were run to examine the unique impact of BAS-2 scores (Block 2) on dependent variables (i.e., depression symptoms, anxiety symptoms) after first controlling for all other demographics and background measures (Block 1) given the large sample sizes. Since two different regression analyses were performed, a Bonferroni correction was used for these analyses, based on the following value, *P* < 0.05 / 2 (two tailed) (64). Similarly, hierarchical binary logistic regression analyses were run to test the extent to which BAS-2 scores (Block 2) were associated with suicidality (yes vs. no) in the entire sample and within each gender, after first controlling for all other demographic/background measures. The significance level was set at the conventional *P* < 0.05 (two-tailed) for these analyses.

## Results

### Descriptive Data for the Sample

Of 2,282 college students who were initially invited, 80 students refused to participate, yielding a response rate of 96.49%. From 2,202 college students who completed the survey, data of 144 participants were excluded due to reporting the presence of major physical and/or psychiatric conditions. Finally, 2,058 college students (1,393 women, 665 men) were included for analyses (Table 1). Average PHQ-9 and GAD-7 scores of 5.51 ± 5.44 and 4.00 ± 4.77, respectively, in our whole sample, were similar to average scores in another sample of healthy Chinese college students (PHQ-9: 5.65 ± 5.11, GAD-7: 3.79 ± 4.08) (65) and suggested levels of these psychiatric symptoms were comparable to rates typically found in China-based college student studies.

**Table 1:** Socio-demographical and clinical characteristics of the whole sample and by gender.

**Variable**	**Total**		**Women**		**Men**		**Univariate analyses**		
	**(*****N*** **=** **2,058)**		**(*****N*** **=** **1,393)**		**(*****N*** **=** **665)**				
	***N***	**%**	***N***	**%**	***N***	**%**	**χ^2^/*F***	**df**	***P***
Academic grade	–	–	–	–	–	–	–	–	–
First year	1,337	65.0	881	65.9	456	68.6	29.46	4	**<0.001**
Second year	393	19.1	300	21.5	93	14.0			
Third year	172	8.4	120	8.6	52	7.8			
Fourth year	79	3.8	55	3.9	24	3.6			
Fifth year	77	3.7	37	2.7	40	6.0			
Health-related major	1,158	56.3	762	54.7	396	59.5	4.30	1	**0.038**
Residence (rural)	1,229	59.7	810	58.1	583	41.9	4.42	1	**0.036**
Perceived health status	–	–	–	–	–	–	–	–	**–**
Poor/Fair^a^	478	23.2	339	24.3	139	20.9	2.98	1	0.084
Good	1,580	76.8	1,054	75.7	526	79.1			
Suicidality	168	8.2	114	8.2	54	8.1	0.00	1	0.961
**Variable**	**Mean**	**SD**	**Mean**	**SD**	**Mean**	**SD**	***t/Z***	**df**	***P***
Age (years)	19.89	2.17	19.76	2.05	20.16	2.36	−3.96	2056	**<0.001**
BAS-2 score	3.53	0.88	3.47	0.85	3.64	0.91	−4.14	2056	**<0.001**
Fatigue total	3.91	2.37	4.03	2.33	3.65	2.44	−3.57	–^b^	**<0.001**
Pain total	1.77	2.03	1.79	2.00	1.72	2.09	−1.10	–^b^	0.273
PHQ-9 total	5.51	5.44	5.75	5.32	5.01	5.65	−4.28	–^b^	**<0.001**
GAD-7 total	4.00	4.77	4.37	4.77	3.23	4.672	−6.81	–^b^	**<0.001**
Body mass index	23.52	7.74	23.17	7.67	24.25	7.86	−4.68	–^b^	**<0.001**

*Bolded values, P < 0.05; SD, standard deviation*.

a *Due to the low percentage of poor health status, the poor and fair health status was pooled*;

b *Mann-Whitney U Test; BAS-2, Body Appreciation Scale-2; GAD-7, Generalized Anxiety Disorder-7; PHQ-9, the 9-item Patient Health Questionnaire*.

### Body Appreciation Differences Between College Women and Men

The average BAS-2 score for the total sample was 3.53 ± 0.875. A two-sample t test indicated college men had a significantly higher average BAS-2 score than did college women (3.64 ± 0.91 vs. 3.47 ± 0.85, *P* < 0.001, df = 2,056, Cohen's d = 0.193). Other univariate analyses found significant differences between women vs. men on age (*P* < 0.001), academic grade (*P* < 0.001), study major (*P* = 0.038), residence (*P* = 0.036), severity of fatigue (*P* < 0.001), severity of depressive symptoms (*P* < 0.001), severity of anxiety symptoms (*P* < 0.001) and BMI (*P* < 0.001) (Table 1). After controlling for all potential covariates, an ANCOVA found that men continued to report a significantly higher mean BAS-2 score than women did [*F*_(1,2058)_ = 13.244, *P* < 0.001].

### Associations of Body Appreciation With Depression Symptoms, Anxiety Symptoms, and Suicidality

Within the entire sample, hierarchical multiple regression analyses indicated BAS-2 scores had significant, unique, inverse associations with PHQ-9 total scores (β = −0.129, *R*^2^ change = 0.014, *P* < 0.001) and GAD-7 total scores (β = −0.101, *R*^2^ change = 0.009, *P* < 0.001), after controlling for all demographics and measures of background functioning. Among college women, BAS-2 scores also had significant, unique, inverse relations with PHQ-9 total scores (β = −0.172, *R*^2^ change = 0.025, *P* < 0.001) and GAD-7 total scores (β = −0.131, *R*^2^change = 0.015, *P* < 0.001) after controlling for all demographics/background factors; as such, young women who reported elevations in BA tended to report lower severity of depression and anxiety. In contrast, BAS-2 scores of college men did not make unique contributions to the prediction of lower PHQ-9 scores (β = −0.049, *R*^2^ change = 0.002, *P* = 0.875) or lower GAD-7 scores (β = −0.043, *R*^2^ change = 0.002, *P* = 0.249) (Table 2).

**Table 2:** ^d^Unique associations of BAS-2 scores with depression, anxiety, and suicidality in the total sample and by gender after controlling for demographics and background measures (see the complete hierarchical regression results in Supplementary Table 1).

**1. Hierarchical multiple linear regression with depressive symptoms as the dependent variable** ^**b**^																		
**Variables**	**Whole sample** ^**a**^						**Women**						**Men**					
	**R**^**2**^ **Change**	**B**	***P***	***β***	**97.5% CI of B**		**R**^**2**^ **Change**	**B**	***P***	***β***	**97.5% CI of B**		**R**^**2**^ **Change**	**B**	***P***	***β***	**97.5% CI of B**	
BAS-2	0.014	−0.804	**<0.001**	−0.129	−1.087	−0.520	0.025	−1.079	**<0.001**	−0.172	−1.424	−0.733	0.002	−0.305	0.875	−0.049	−0.188	0.216
BMI, academic grade, major, residence, perceived health status, age, fatigue, and pain were controlled for covariates in this model																		
**2. Hierarchical multiple linear regression with anxiety symptoms as the dependent variable** ^**b**^																		
**Variables**	**Whole sample** ^**a**^						**Women**						**Men**					
	**R**^**2**^ **Change**	**B**	***P***	***β***	**97.5% CI of B**		**R**^**2**^ **Change**	**B**	***P***	***β***	**97.5% CI of B**		**R**^**2**^ **Change**	**B**	***P***	***β***	**97.5% CI of B**	
BAS-2	0.009	−0.549	**<0.001**	−0.101	−0.803	−0.296	0.015	−0.733	**<0.001**	−0.131	−1.048	−0.417	0.002	−0.22	0.249	−0.043	−0.65	0.209
BMI, academic grade, major, residence, perceived health status, age, fatigue, and pain were controlled for covariates in this model																		
**3. Hierarchical multiple logistic regression with suicidality as dependent variable** ^**c**^																		
**Variables**		**Whole sample** ^**a**^						**Women**						**Men**				
		**OR**	***P***	**95% CI**				**OR**	***P***	**95% CI**				**OR**	***P***	**95% CI**		
BAS-2		0.788	**0.020**	0.644	0.963			0.639	**0.001**	0.491	0.832			1.178	0.345	0.839	1.654	
BMI, academic grade, major, residence, perceived health status, age, fatigue, pain, PHQ-9, and GAD-7 were controlled for covariates in this model																		

*PHQ-9, the 9-item Patient Health Questionnaire; BMI, Body Mass Index; BAS-2, Body Appreciation Scale-2; GAD-7, Generalized Anxiety Disorder-7; CI, confidence interval*;

a *Gender was controlled as covariate in the whole sample regression analyses*;

b *P-value was set at <0.025 in the depressive and anxiety model due to the Bonferroni correction*;

c *P-value was set at <0.05 in the suicidality model*;

d *To save space, this table only shows the specific BAS-2 values in Block 2, the complete results of hierarchical regression analyses could be seen in Supplementary Table 1. Bold values highlighted the P values which are statistically significant*.

In multiple logistic regression analyses, lower BAS-2 scores emerged as a significant predictor of the presence of suicidality in the entire sample (OR = 0.788, *P* = 0.020, 95% CI = 0.644–0.963) and within the subsample of women (OR = 0.639, *P* = 0.001, 95% CI = 0.491–0.832) but not among the men (OR = 1.178, *P* = 0.345) (Table 2). Supplementary Table 1 provides complete regression analysis results.

## Discussion

This study is the first to assess gender differences in BA within a national college student sample from China in addition to exploring links of BA with commonly reported psychiatric symptoms and suicidality within each gender independent of various demographics and background characteristics. We discuss the main findings related to these purposes below in addition to their potential implications for research and practice.

### Gender Differences in Body Appreciation

In support of the initial hypothesis, Chinese college women reported a significantly less BA, on average, than Chinese college men did; the difference was highly significant though the associated effect size was small (Cohen's d = 0.193; *P* < 0.001). Notably, the BA difference between women and men remained highly significant even after statistically controlling for significant gender differences on demographics (age, BMI, study major, academic grade, residence), perceived health status, and current severity of commonly reported psychiatric and somatic symptoms (i.e., depressive symptoms, anxiety symptoms, fatigue, pain). As such, the gender difference in BA observed in our sample is consistent with results from previous college student studies (10, 66, 67) as well as a meta-analysis of 40 studies indicating university women generally report less BA than university men do (14). Also in line with general psychiatry references and China-based epidemiological studies on gender differences in psychiatric symptoms, women in our sample reported significantly higher severities of depressive symptoms, anxiety symptoms and fatigue (65, 68–71), hypothesized to result from gender differences in various biological and psychosocial factors (72–74). Notably, however, the gender difference in BA found in this study could not be explained by corresponding links between gender and these psychiatric disturbances or specific demographics that were statistically controlled in associated analysis.

Instead, experiences with key facets of BA (i.e., approval, acceptance, respect, protection of the body, rejection of unrealistic, media-promoted appearance ideals) may help to explain this gender difference. For example, Fredrickson and Roberts (75) assert that gender socialization routinely involves sexual objectification from the broader society wherein women's identities and worth are shaped by how well they conform to a narrowly defined appearance ideal based on ultra-thinness. Sexual objectification of girls and women can lead, ultimately, to self-objectification wherein one treats the self as an object of scrutiny, engages in persistent body surveillance and/or frequently compares her body to the ultra-thin feminine ideal (76). High levels of self-objectification lead to anxiety about appearance and physical safety, reduced awareness of body sensations, and heightened body shame (77–79). These premises were tested initially among women in Western countries but there is mounting evidence that sexual objectification and self-objectification are also more highly salient for college-age Chinese women than their male peers (15, 24, 80, 81). As such, young Chinese women whose physical appearance deviates from the culturally sanctioned feminine ideal may have more difficulty accepting and appreciating their body. Conversely, young Chinese men may have comparatively high BA levels because they are less likely to define themselves through conformity to a masculine physical appearance ideal than non-physical attributes (82).

Similarly, in line with sociocultural models of body image (83), adolescent and young adult Chinese women tend to experience more appearance-related social pressure from traditional mass media and social media than their male counterparts do (84–87). Because of gender differences in such pressure (e.g., to lose weight or be thinner, to have particular constelations of facial features), young Chinese women may be more prone to focus on their body's appearance than their body's needs (e.g., dieting to being thinner instead of eating to be healthy). More generally, the “other-directed” orientation more typically adopted among young Chinese women than young Chinese men may reinforce a propensity to ignore the body's needs. To elaborate, some authors contend that Chinese women are more often socialized to sacrifice for their family and society (88). This moral constraint might contribute to feelings of shame or accusations of selfishness that inhibit women's care for their own body's needs compared to the needs of other family members.

### Relations Between Body Appreciation and Psychiatric Symptoms

Regarding the second main study focus on relations between BA and psychiatric symptoms, higher BAS-2 scores were associated with lower levels of reported depressive symptoms, anxiety symptoms and suicidality in the overall sample and the subsample of women but not among men. Once again, these associations could not be explained by demographics aside from gender or measures of background functioning which had been statistically controlled in analyses. Findings for the entire sample aligned with our predictions and previous studies linking elevations in BA to lower levels of depression and anxiety (16–21). However, with only one exception (17), past studies did not compare correlations between BA and psychiatric symptoms for women vs. men. Consequently, independent assessments of these associations within each gender were a relatively novel facet of this research.

Significant links between BA and symptoms of depression and anxiety among women but not men were not likely due to the smaller number of men (*N* = 665) than women (*N* = 1,393 women) in our sample. That is, although sample sizes do affect *p* values, they have little bearing on effect size strengths. In our sample, strengths of relation between BA and psychiatric symptoms were approximately three times stronger among women than among men (see Table 2). Furthermore, in prediction models for women, BA accounted for modest, unique, statistically significant variance in psychiatric symptoms beyond the impact of other research measures. Conversely, in prediction models for men, unique contributions of BA were negligible and not statistically significant.

Instead, select gender-based sociocultural models and neuroscience frameworks may offer more plausible hypotheses that account for stronger links of BA with symptoms of depression and anxiety for women than for men. For example, gender roles theorists posit that women more typically use their physical attractiveness to gain social resources and social status in patriarchal societies; conversely, men have increased social and economic power due to their status as breadwinners and exchange their social power for women's beauty (89, 90). Consequently, personal traits and abilities are more highly valued in men while physical appearance is emphasized more strongly for women (91). Thus, women's attitudes toward their own body (both negative and positive) have stronger links with personal self-worth and mental health than men's body image attitudes do. Similarly, proponents of models that emphasize sexual objectification contend that these processes reduce a woman's worth to her sexual functioning and how well she conforms to a thin standard of feminine beauty (92). Sexual objectification can exacerbate mental health problems that disproportionately affect women (i.e., depression, anxiety, eating disorders) through directly related trauma experiences (e.g., rape, physical or sexual assault) and indirectly related experiences including appearance social pressure, internalized societal beauty standards and self-objectification. These sociocultural factors may also have links with gender differences in biological responses to external physical appearance cues. For example, Shirao et al. (93) found that exposure to body image stimuli (i.e., words to describe one's body) were linked to increased cognitive network responsiveness and emotional network responsiveness, respectively among men and women. One hypothesis that follows is that enhanced emotion region responsiveness to such stimuli among women strengthens associations between body image experiences and emotional distress; conversely, increased cognitive network activity in response to these cues among men attenuates body image–distress associations.

Together sociocultural and biological factors may help to explain stronger links of BA with depressive and anxiety symptoms, in general, for women than men. However, results also underscore how individual differences in positive body image experiences such as BA may be particularly important for women as factors that offer protection against depression and anxiety. Aside from being less susceptible to effects of sexual objectification and definitions of identity based on limiting gender role prescriptions, women who have higher levels of BA also tend to have more optimistic orientations toward the future, proactive styles of coping, and awareness/trust of their body's internal hunger and satiety cues as guides for eating (9, 10).

To our knowledge, this study is also the first to document significant associations between higher BA levels and reduced suicidality in the overall sample and among women. This finding converges with three lines of related work. First, past research has reported negative body image predicts suicidal ideation among college students, above and beyond effects of depression, hopelessness, and past suicidal behaviors (94). Similarly, suicidal adolescents report more negative body image, less body protection, and heightened body aberration compared to non-suicidal adolescents (95). Third, as noted in the introduction, an array of studies has documented positive associations between BA and a variety of health-protective behaviors that promote longevity (16, 35–37). In tandem with these studies, our findings provide foundations for the hypothesis that enhanced BA levels protect against suicidality by fostering general self-care (96) and buffering against disregard for one's body and a propensity toward self-harm engendered by negative body image (13, 97, 98).

An alternative hypothesis for BA-suicidality results follows from emotion regulation perspectives. On one hand, young women who have high levels of BA more often use positive emotion regulation strategies (i.e., proactive coping, rational acceptance of appearance, cognitive reappraisal) that correlate with lower levels of emotional disturbances (99). Conversely, young women who experience low levels of BA may be prone to adopting emotion dysregulation strategies (e.g., rumination, emotion suppression, avoidant coping) implicated in emotional disturbances, suicidal desire and engagement in non-suicidal self-injury behaviors (100). Direct tests of within gender associations between BA, emotion regulation, body image threats or concerns, and suicidality are a potentially important line of future research in the field.

Finally, in contrast to significant relations with psychiatric symptoms observed among college women, BA had noticeably weaker, non-significant associations with severity of depression symptoms, anxiety symptoms, and suicidality among men in this study. These results contrast with significant, inverse correlations between BA and depression found within both samples of U.S. university men and women (17). Methodological factors (e.g., cultural differences between samples, the use of different measures) may have contributed to inconsistent results for men between these two studies. Nonetheless, most available evidence examining links between BA and psychiatric symptoms has focused exclusively upon women. Hence, there is a clear need for more studies that include men in sampling before conclusions can be drawn about the robustness of associations between BA and measures of depression, anxiety, and suicidality for women vs. men.

### Implications for Research and Practice

In relation to implications, to date, most China-based studies on body image have focused on correlates of or risk factors for negative body image (26, 80, 81). This study provides foundations for extensions examining links of BA or other facets of positive body image with gender, psychiatric disturbances and suicidality in China and other understudied cultures. Theories of negative body image (78, 83, 101), have tended to guide targets for treatment and prevention with an emphasis on reducing risk factors such as sexual objectification, internalization of unrealistic appearance ideals, and effects of appearance-related media. Undoubtedly, these are important foci for interventions designed to reduce damaging, potentially life-threatening effects of negative body image.

However, this study underscores the potential value of *also* considering facets of positive body image as a means of reducing psychiatric disturbances and suicide risk, particularly among young women. Clinical strategies that bolster facets of BA (e.g., increasing awareness and respect for one's body, appreciating diverse expressions of beauty) help to reduce distress and enhance positive mental health (12, 102–105). For example, in a directly relevant randomized control trial (106), young Chinese women were assigned to a widely used dissonance-based eating disorder prevention intervention (107) vs. an education brochure control condition. Compared to controls, treatment group women experienced significantly sharper increases in BA and self-esteem as well as sharper decreases in disordered eating and related risk factors (body dissatisfaction, thin ideal internalization, depression) at a 6-month follow-up. Although dissonance prevention programs were first developed to reduce eating disorder risk factors, module content includes self-affirmation exercises, behavior challenges, and body activism activities designed to encourage positive body image experiences such as self-acceptance, body acceptance, and the pursuit of activities previously avoided because of appearance anxiety or shame (108). Consequently, eating disorder prevention programs merit more attention as approaches that also foster BA and other aspects of positive body image in tandem with reducing risk factors for negative body image.

### Strengths and Limitations

Strengths of this study included its assessment of a large national sample from a comparatively understudied cultural context, comparisons of both gender differences in BA and within gender associations of BA with common psychiatric symptoms, and its novel findings regarding relations between BA and suicidality. Strengths aside, several limitations are also apparent. First, because the sample comprised Chinese college students, findings may not apply to college students in other countries or different age groups. Second, because the suicidality index we used assessed both ideation and planning, future studies with separate items tapping each experience are needed to disentangle associations with BA more precisely. Third, because assessments were based solely on self-reports, certain response biases (e.g., social desirability, random responding) cannot be ruled out as influences on findings. For example, mandatory responses were required for the online survey to reduce missing data. Although we did not detect out of range values that might flag random responding, “attention check” items should be included in future studies to provide additional quality checks for response biases. Finally, while BA is a central facet of positive body image, other facets such as body functionality should be incorporated within future work to provide a more complete understanding of links between gender, positive body image, and psychiatric disturbances.

## Conclusion

In sum, this study found Chinese college women had a significantly lower average level of BA than did Chinese college men. Within gender analyses also revealed elevations in BA were related to significantly lower levels of depressive symptoms, anxiety symptoms and suicidality among women but not among men. In light of these findings, interventions that increase BA should be tested more widely among college students, particularly undergraduate women, given that such strategies could mitigate effects of damaging sociocultural influences, decrease disturbances in eating, and promote positive body image in addition to reducing common psychiatric symptoms and suicidality.

## Data Availability Statement

The Institutional Review Board (IRB) of Beijing Anding Hospital that approved the study prohibits the authors from making the research dataset of clinical studies publicly available. Readers and all interested researchers may contact Dr. Rui Liu (Email address: liuruicomeon@163.com) for details. Dr. Liu will apply to the IRB of Beijing Anding Hospital for the release of the data.

## Ethics Statement

The studies involving human participants were reviewed and approved by the Institutional Review Board (IRB) of Beijing Anding Hospital. Written informed consent to participate in this study was provided by the participants' legal guardian/next of kin.

## Author Contributions

Z-HL and Y-TX: study design. Z-HL, HC, WB, SL, HL, XC, HQ, and RL: data collection and analysis and interpretation. Z-HL, TC, TJ, and Y-TX: drafting of the manuscript. TJ: critical revision of the manuscript. All authors approval of the final version for publication.

## Funding

The study was supported by the National Science and Technology Major Project for Investigational New Drug (2018ZX09201-Q16014), the Beijing Hospitals Authority Clinical Medicine Development of special funding support [XMLX202128], the University of Macau (MYRG2019-00066-FHS), Suzhou Key Medical Center for Psychiatric Diseases (Szzx201509), and the Chinese National Natural Science Foundation (#31871141).

## Conflict of Interest

The authors declare that the research was conducted in the absence of any commercial or financial relationships that could be construed as a potential conflict of interest.

## Publisher's Note

All claims expressed in this article are solely those of the authors and do not necessarily represent those of their affiliated organizations, or those of the publisher, the editors and the reviewers. Any product that may be evaluated in this article, or claim that may be made by its manufacturer, is not guaranteed or endorsed by the publisher.
