# Supplementary material for: Gender Differences in Body Appreciation and Its Associations With Psychiatric Symptoms Among Chinese College Students: A Nationwide Survey
Source: Front Psychiatry. 2022 Feb 17;13:771398. doi: 10.3389/fpsyt.2022.771398 (PMC8892204; doi:10.3389/fpsyt.2022.771398)
Supplement: Supplementary file 1 [file Table_1.DOCX]

**Supplementary Table 1: Complete version of hierarchical regression analyses**

| 1. **Hierarchical multiple linear regression with depressive symptoms as the dependent variable^b^** | | | | | | | | | | | | | | | | | | | | | | | | | | |
| --- | --- | --- | --- | --- | --- | --- | --- | --- | --- | --- | --- | --- | --- | --- | --- | --- | --- | --- | --- | --- | --- | --- | --- | --- | --- | --- |
| **Variables** |  | | | **Whole sample** | | | | | |  | **Women** | | | | | **Men** | | | | | | | | | | |
|  | **B** | ***β*** | | | ***P*** | | **97.5% CI of B** | | | **B** | ***β*** | ***P*** | **97.5% CI of B** | | | **B** | | ***β*** | | | ***P*** | | **97.5% CI of B** | | | |
| **Block 1** | | | | | | | | | | | | | | | | | | | | | | | | | | |
| Age | .017 | 0.007 | | | **<0.001** | | 2.021 | | 7.374 | 0.011 | 0.004 | 0.865 | -0.137 | | 0.159 | 0.013 | 0.005 | | | 0.888 | | | | -0.189 | | 0.215 |
| Gender^a^ | -.353 | -0.030 | | | 0.744 | | -0.101 | | 0.136 | - | - | - | - | | - | - | - | | | - | | | | - | | - |
| Academic grade | -.281 | -0.054 | | | 0.113 | | -0.853 | | 0.146 | -0.274 | -0.051 | 0.051 | -0.588 | | 0.040 | -0.315 | -0.064 | | | 0.088 | | | | -0.729 | | 0.100 |
| Major | .480 | 0.044 | | | **0.011** | | -0.530 | | -0.032 | 0.694 | 0.065 | **0.008** | 0.107 | | 1.281 | -0.047 | -0.004 | | | 0.910 | | | | -0.986 | | 0.892 |
| Residence | -.015 | -0.001 | | | 0.030 | | -0.017 | | 0.977 | 0.054 | 0.005 | 0.834 | -0.526 | | 0.635 | -0.178 | -0.015 | | | 0.658 | | | | -1.084 | | 0.727 |
| Perceived health status | -1.625 | -0.126 | | | 0.947 | | -0.502 | | 0.473 | -1.596 | -0.129 | **<0.001** | -2.264 | | -0.927 | -1.750 | -0.126 | | | **<0.001** | | | | -2.840 | | -0.660 |
| Fatigue | .787 | 0.343 | | | **<0.001** | | -2.196 | | -1.055 | 0.803 | 0.352 | **<0.001** | 0.666 | | 0.940 | 0.755 | 0.326 | | | **<0.001** | | | | 0.541 | | 0.970 |
| Pain | .481 | 0.179 | | | **<0.001** | | 0.671 | | 0.902 | 0.441 | 0.166 | **<0.001** | 0.278 | | 0.604 | 0.557 | 0.206 | | | **<0.001** | | | | 0.307 | | 0.807 |
| BMI | .015 | 0.021 | | | **<0.001** | | 0.345 | | 0.617 | 0.028 | 0.041 | 0.083 | -0.008 | | 0.064 | -0.015 | -0.021 | | | 0.539 | | | | -0.069 | | 0.039 |
| **Block 2** | | | | | | | | | | | | | | | | | | | | | | | | | | |
| Age | 0 | 0 | | | 0.994 | | -0.117 | | 0.118 | -0.029 | -0.011 | 0.654 | -0.175 | | 0.117 | 0.014 | 0.006 | | | 0.875 | | | | -0.188 | | 0.216 |
| Gender^a^ | -0.235 | -0.020 | | | 0.289 | | -0.732 | | 0.262 | - | - | - | - | | - | - | - | | | - | | | | - | | - |
| Academic grade | -0.227 | -0.043 | | | 0.040 | | -0.474 | | 0.020 | -0.173 | -0.032 | 0.211 | -0.484 | | 0.137 | -0.306 | -0.062 | | | 0.097 | | | | -0.721 | | 0.108 |
| Major | 0.290 | 0.026 | | | 0.191 | | -0.207 | | 0.787 | 0.454 | 0.042 | 0.081 | -0.129 | | 1.036 | -0.124 | -0.011 | | | 0.769 | | | | -1.071 | | 0.823 |
| Residence | -0.003 | 0.000 | | | 0.989 | | -0.486 | | 0.480 | 0.045 | 0.004 | 0.858 | -0.525 | | 0.616 | -0.163 | -0.014 | | | 0.686 | | | | -1.068 | | 0.742 |
| Perceived health status | -1.390 | -0.108 | | | **<0.001** | | -1.961 | | -0.820 | -1.318 | -0.106 | **<0.001** | -1.981 | | -0.655 | -1.636 | -0.118 | | | **0.001** | | | | -2.742 | | -0.531 |
| Fatigue | 0.755 | 0.329 | | | **<0.001** | | 0.640 | | 0.870 | 0.757 | 0.332 | **<0.001** | 0.622 | | 0.893 | 0.746 | 0.322 | | | **<0.001** | | | | 0.531 | | 0.961 |
| Pain | 0.419 | 0.156 | | | **<0.001** | | 0.282 | | 0.556 | 0.361 | 0.136 | **<0.001** | 0.198 | | 0.523 | 0.532 | 0.196 | | | **<0.001** | | | | 0.279 | | 0.785 |
| BMI | 0.006 | 0.009 | | | 0.645 | | -0.024 | | 0.036 | 0.015 | 0.021 | 0.359 | -0.021 | | 0.051 | -0.017 | -0.024 | | | 0.483 | | | | -0.071 | | 0.037 |
| BAS-2 | -0.804 | -0.129 | | | **<0.001** | | -1.087 | | -0.520 | -1.079 | -0.172 | **<0.001** | -1.424 | | -0.733 | -0.305 | -0.049 | | | 0.875 | | | | -0.188 | | 0.216 |
| **Model Summary** | **R^2^** | **Adjusted R^2^** | | | **R^2^ Change** | | **F Change** | | **Sig. F Change** | **R^2^** | **Adjusted R^2^** | **R^2^ Change** | **F Change** | | **Sig. F Change** | **R^2^** | **Adjusted R^2^** | | | **R^2^ Change** | | | | **F Change** | | **Sig. F Change** |
| Model 1 | 0.263 | 0.260 | | | 0.263 | | 81.222 | | **<0.001** | 0.258 | 0.253 | 0.258 | 59.998 | | **<0.001** | 0.271 | 0.262 | | | 0.271 | | | | 30.486 | | **<0.001** |
| Model 2 | 0.277 | 0.274 | | | 0.014 | | 40.468 | | **<0.001** | 0.283 | 0.287 | 0.025 | 49.062 | | **<0.001** | 0.273 | 0.263 | | | 0.002 | | | | 1.890 | | 0.170 |
| **2. Hierarchical multiple linear regression with anxiety symptoms as the dependent variable^b^** | | | | | | | | | | | | | | | | | | | | | | | | | | |
| **Variables** |  | | | **Whole sample** | | | | | |  | **Women** | | | | | **Men** | | | | | | | | | | |
|  | **B** | ***β*** | | | ***P*** | | **97.5% CI of B** | | | **B** | ***β*** | ***P*** | **97.5% CI of B** | | | **B** | | ***β*** | | | ***P*** | | **97.5% CI of B** | | | |
| **Block 1** | | | | | | | | | | | | | | | | | | | | | | | | | | |
| Age | 0.052 | 0.024 | | | 0.269 | | -0.054 | | 0.158 | 0.038 | 0.016 | 0.529 | -0.096 | | 0.172 | 0.053 | 0.027 | | | 0.492 | | | | -0.121 | | 0.227 |
| Gender^a^ | -0.802 | -0.079 | | | **<0.001** | | -1.247 | | -0.356 | - | - | - | - | | - | - | - | | | - | | | | - | | - |
| Academic grade | -0.234 | -0.051 | | | **0.018** | | -0.456 | | -0.012 | -0.232 | -0.048 | 0.068 | -0.516 | | 0.053 | -0.253 | -0.062 | | | 0.112 | | | | -0.61 | | 0.104 |
| Major | 0.196 | 0.020 | | | 0.321 | | -0.247 | | 0.640 | 0.377 | 0.039 | 0.112 | -0.156 | | 0.91 | -0.224 | -0.024 | | | 0.534 | | | | -1.033 | | 0.585 |
| Residence | 0.022 | 0.002 | | | 0.908 | | -0.413 | | 0.457 | 0.008 | 0.001 | 0.974 | -0.519 | | 0.534 | 0.039 | 0.004 | | | 0.911 | | | | -0.741 | | 0.819 |
| Perceived health status | -1.306 | -0.116 | | | **<0.001** | | -1.814 | | -0.797 | -1.283 | -0.115 | **<0.001** | -1.889 | | -0.676 | -1.433 | -0.125 | | | **0.001** | | | | -2.373 | | -0.494 |
| Fatigue | 0.707 | 0.352 | | | **<0.001** | | 0.604 | | 0.810 | 0.778 | 0.38 | **<0.001** | 0.654 | | 0.902 | 0.563 | 0.294 | | | **<0.001** | | | | 0.378 | | 0.748 |
| Pain | 0.312 | 0.133 | | | **<0.001** | | 0.190 | | 0.433 | 0.293 | 0.123 | **<0.001** | 0.145 | | 0.441 | 0.363 | 0.162 | | | **<0.001** | | | | 0.148 | | 0.578 |
| BMI | -0.005 | -0.008 | | | 0.698 | | -0.031 | | 0.022 | 0.002 | 0.004 | 0.865 | -0.03 | | 0.035 | -0.019 | -0.031 | | | 0.371 | | | | -0.065 | | 0.028 |
| **Block 2** | | | | | | | | | | | | | | | | | | | | | | | | | | |
| Age | 0.041 | 0.018 | | | 0.387 | | -0.065 | | 0.146 | 0.01 | 0.004 | 0.864 | -0.123 | | 0.144 | 0.054 | 0.027 | | | 0.483 | | | | -0.12 | | 0.228 |
| Gender^a^ | -0.721 | -0.071 | | | **<0.001** | | -1.166 | | -0.276 | - | - | - | - | | - | - | - | | | - | | | | - | | - |
| Academic grade | -0.197 | -0.043 | | | 0.046 | | -0.419 | | 0.024 | -0.163 | -0.034 | 0.197 | -0.447 | | 0.12 | -0.247 | -0.061 | | | 0.121 | | | | -0.604 | | 0.111 |
| Major | 0.066 | 0.007 | | | 0.738 | | -0.379 | | 0.511 | 0.214 | 0.022 | 0.368 | -0.319 | | 0.746 | -0.279 | -0.029 | | | 0.442 | | | | -1.096 | | 0.537 |
| Residence | 0.030 | 0.003 | | | 0.875 | | -0.402 | | 0.463 | 0.002 | 0 | 0.994 | -0.52 | | 0.523 | 0.05 | 0.005 | | | 0.886 | | | | -0.73 | | 0.83 |
| Perceived health status | -1.145 | -0.101 | | | **<0.001** | | -1.656 | | -0.634 | -1.094 | -0.098 | **<0.001** | -1.7 | | -0.488 | -1.351 | -0.118 | | | **0.002** | | | | -2.304 | | -0.398 |
| Fatigue | 0.686 | 0.341 | | | **<0.001** | | 0.583 | | 0.788 | 0.747 | 0.365 | **<0.001** | 0.623 | | 0.871 | 0.556 | 0.29 | | | **<0.001** | | | | 0.371 | | 0.741 |
| Pain | 0.270 | 0.115 | | | **<0.001** | | 0.147 | | 0.392 | 0.238 | 0.1 | **<0.001** | 0.09 | | 0.387 | 0.345 | 0.154 | | | **<0.001** | | | | 0.127 | | 0.563 |
| BMI | -0.011 | -0.017 | | | 0.379 | | -0.037 | | 0.016 | -0.007 | -0.011 | 0.652 | -0.039 | | 0.026 | -0.02 | -0.034 | | | 0.333 | | | | -0.067 | | 0.027 |
| BAS-2 | -0.549 | -0.101 | | | **<0.001** | | -0.803 | | -0.296 | -0.733 | -0.131 | **<0.001** | -1.048 | | -0.417 | -0.22 | -0.043 | | | 0.249 | | | | -0.65 | | 0.209 |
| **Model Summary** | **R^2^** | **Adjusted R^2^** | | | **R^2^ Change** | | **F Change** | | **Sig. F Change** | **R^2^** | **Adjusted R^2^** | **R^2^ Change** | **F Change** | | **Sig. F Change** | **R^2^** | **Adjusted R^2^** | | | **R^2^ Change** | | | | **F Change** | | **Sig. F Change** |
| Model 1 | 0.237 | 0.234 | | | 0.237 | | 70.809 | | **<0.001** | 0.242 | 0.237 | 0.242 | 55.097 | | **<0.001** | 0.207 | 0.198 | | | 0.207 | | | | 21.465 | | **<0.001** |
| Model 2 | 0.246 | 0.242 | | | 0.009 | | 23.580 | | **<0.001** | 0.256 | 0.251 | 0.015 | 22.076 | | **<0.001** | 0.209 | 0.198 | | | 0.002 | | | | 1.332 | | 0.249 |
| **3. Hierarchical multiple logistic regression with suicidality as dependent variable^c^** | | | | | | | | | | | | | | | | | | | | | | | | | | |
| **Variables** |  | | | **Whole sample** | | | | | |  | **Women** | | | | | **Men** | | | | | | | | | | |
|  | **OR** | | ***P*** | | | **95% CI** | | | | **OR** | ***P*** | **95% CI** | | | | **OR** | | | ***P*** | | | **95% CI** | | | | |
| **Block 1** | | | | | | | | | | | | | | | | | | | | | | | | | | |
| Age | 1.003 | | 0.943 | | | 0.912 | | 1.104 | | 1.01 | 0.866 | 0.898 | | 1.136 | | 1 | | | 1 | | | 0.842 | | | 1.187 | |
| Gender^a^ | 1.162 | | 0.425 | | | 0.804 | | 1.679 | | - | - | - | | - | | - | | | - | | | - | | | - | |
| Academic grade | 1.109 | | 0.289 | | | 0.916 | | 1.343 | | 0.939 | 0.634 | 0.723 | | 1.219 | | 1.468 | | | **0.011** | | | 1.094 | | | 1.971 | |
| Major | 2.334 | | **<0.001** | | | 1.615 | | 3.374 | | 1.716 | **0.016** | 1.105 | | 2.666 | | 5.016 | | | **<0.001** | | | 2.428 | | | 10.362 | |
| Residence | 1.189 | | 0.336 | | | 0.835 | | 1.694 | | 1.684 | **0.018** | 1.094 | | 2.592 | | 0.556 | | | 0.091 | | | 0.281 | | | 1.099 | |
| Perceived health status | 0.986 | | 0.943 | | | 0.674 | | 1.443 | | 0.863 | 0.519 | 0.55 | | 1.353 | | 1.307 | | | 0.481 | | | 0.62 | | | 2.754 | |
| Fatigue | 1.193 | | **<0.001** | | | 1.092 | | 1.303 | | 1.199 | **0.001** | 1.073 | | 1.339 | | 1.216 | | | **0.013** | | | 1.042 | | | 1.421 | |
| Pain | 1.083 | | 0.067 | | | 0.995 | | 1.18 | | 1.098 | 0.079 | 0.989 | | 1.218 | | 1.046 | | | 0.573 | | | 0.895 | | | 1.223 | |
| PHQ-9 | 1.104 | | **<0.001** | | | 1.057 | | 1.154 | | 1.139 | **<0.001** | 1.079 | | 1.202 | | 1.044 | | | 0.29 | | | 0.964 | | | 1.131 | |
| GAD-7 | 1.007 | | 0.767 | | | 0.959 | | 1.058 | | 0.992 | 0.798 | 0.935 | | 1.053 | | 1.048 | | | 0.321 | | | 0.955 | | | 1.15 | |
| BMI | 1 | | 0.988 | | | 0.978 | | 1.022 | | 1.001 | 0.933 | 0.975 | | 1.028 | | 1 | | | 0.984 | | | 0.962 | | | 1.038 | |
| **Block 2** | | | | | | | | | | | | | | | | | | | | | | | | | | |
| Age | 1.002 | | 0.969 | | | 0.911 | | 1.102 | | 1.007 | 0.903 | 0.894 | | 1.135 | | 0.998 | | | 0.986 | | | 0.84 | | | 1.187 | |
| Gender^a^ | 1.208 | | 0.316 | | | 0.835 | | 1.749 | | - | - | - | | - | | - | | | - | | | - | | | - | |
| Academic grade | 1.119 | | 0.253 | | | 0.923 | | 1.356 | | 0.951 | 0.711 | 0.727 | | 1.242 | | 1.468 | | | **0.011** | | | 1.092 | | | 1.973 | |
| Major | 2.251 | | **<0.001** | | | 1.555 | | 3.26 | | 1.62 | **0.034** | 1.038 | | 2.528 | | 5.208 | | | **<0.001** | | | 2.505 | | | 10.827 | |
| Residence | 1.189 | | 0.338 | | | 0.835 | | 1.694 | | 1.629 | **0.028** | 1.056 | | 2.514 | | 0.548 | | | 0.084 | | | 0.277 | | | 1.083 | |
| Perceived health status | 1.04 | | 0.841 | | | 0.708 | | 1.528 | | 0.944 | 0.805 | 0.597 | | 1.493 | | 1.256 | | | 0.554 | | | 0.591 | | | 2.666 | |
| Fatigue | 1.189 | | **<0.001** | | | 1.088 | | 1.299 | | 1.196 | **0.002** | 1.07 | | 1.338 | | 1.22 | | | **0.012** | | | 1.045 | | | 1.424 | |
| Pain | 1.069 | | 0.127 | | | 0.981 | | 1.166 | | 1.071 | 0.204 | 0.964 | | 1.19 | | 1.057 | | | 0.496 | | | 0.902 | | | 1.239 | |
| PHQ-9 | 1.099 | | **<0.001** | | | 1.051 | | 1.148 | | 1.126 | **<0.001** | 1.066 | | 1.189 | | 1.046 | | | 0.271 | | | 0.965 | | | 1.135 | |
| GAD-7 | 1.007 | | 0.781 | | | 0.959 | | 1.058 | | 0.991 | 0.763 | 0.933 | | 1.052 | | 1.047 | | | 0.337 | | | 0.953 | | | 1.15 | |
| BMI | 0.997 | | 0.793 | | | 0.975 | | 1.019 | | 0.996 | 0.767 | 0.969 | | 1.024 | | 1.001 | | | 0.947 | | | 0.964 | | | 1.04 | |
| BAS-2 | 0.788 | | **0.02** | | | 0.644 | | 0.963 | | 0.639 | **0.001** | 0.491 | | 0.832 | | 1.178 | | | 0.345 | | | 0.839 | | | 1.654 | |
| Footnote: PHQ-9: the 9-item Patient Health Questionnaire; QOL: Quality of Life; BMI: Body Mass Index; BAS-2: Body Appreciation Scale-2; GAD-7: Generalized Anxiety Disorder-7. CI: Confidence interval; ^a:^ gender was controlled as covariate only in the whole sample regression analyses; ^b^: *P* value was set at <0.025 in the depression and anxiety model due to the Bonferroni correction; ^c^: P value was set at <0.05 in the suicidality model. | | | | | | | | | | | | | | | | | | | | | | | | | | |
